# Panhypopituitarism Presenting as Myxedema Coma Unveiling Sheehan's Syndrome

**DOI:** 10.1155/crie/1708614

**Published:** 2025-10-11

**Authors:** Mennaallah Eid, Daniel Joseph Toft

**Affiliations:** Division of Endocrinology, Diabetes, and Metabolism, University of Illinois at Chicago, Chicago, Illinois, USA

## Abstract

Myxedema coma (MC) is a rare and life-threatening complication of uncontrolled hypothyroidism. We report a 46-year-old Hispanic female who presented with hemodynamic instability and hyponatremia. She was initially diagnosed with adrenal insufficiency (AI). Further evaluation revealed myxedema features and panhypopituitarism, with partial empty Sella. A thorough history was notable for postpartum hemorrhage 18 years prior and followed by failure of lactation and secondary amenorrhea which is consistent with Sheehan's syndrome. Our case highlights the delayed diagnosis of central hypothyroidism with normal thyroid stimulating hormone levels without checking free thyroxine level and emphasizing the importance of comprehensive evaluation in similar presentations.

## 1. Introduction

Myxedema coma (MC) is a rare complication of extreme decompensated hypothyroidism. It occurs more frequently in primary hypothyroidism than in central hypothyroidism [1], with an estimated mortality rate of 20%–60% [2]. Triggers include infection, surgery, trauma, and prolonged untreated hypothyroidism. Despite its name, coma is an uncommon presentation; instead, most patients present with altered mental status [3]. The diagnosis and management of MC remain a challenging endocrine emergency, requiring a high clinical suspicion [1].

## 2. Case Presentation

A 46-year-old Hispanic female presented to another facility with fatigue and low energy for weeks. She was found to have hemodynamic instability, severe hyponatremia, low random cortisol, and pericardial effusion. Thyroid-stimulating hormone (TSH) was normal. Initial diagnosis was adrenal insufficiency (AI), and she was managed with intravenous (IV) norepinephrine (NE) infusion, IV hydrocortisone (HC) 50 mg every 8 h, and oral fludrocortisone 0.1 mg/day before being transferred to our intensive care unit (ICU).

On examination, body weight was 62 kg, and height was 152 cm. While receiving IV pressors and stress steroid, vital signs were: blood pressure 91/54 millimeters of mercury (mmHg), heart rate 83 beats per minute (bpm), respiratory rate 12 breaths per minute, temperature 99°F, and oxygen saturation 97% on room air. She was obtunded and sleepy, responding slowly to verbal stimuli and disoriented to time. Loss of the outer third of the eyebrows, periorbital edema, slurred and slow speech, dry thickened skin, and decreased deep tendon reflexes' responses were noted. The thyroid exam was unremarkable. Heart sounds were normal, and the lungs were clear to auscultation.

**Figure 1:**
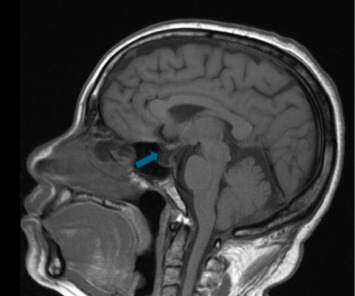
Brain MRI. Sagittal T1 without contrast showing thin enhancing tissue along the floor of the pituitary fossa with cerebrospinal fluid occupying the rest of the fossa suggesting a partially empty sella. Blue arrow points toward empty sella.

**Figure 2:**
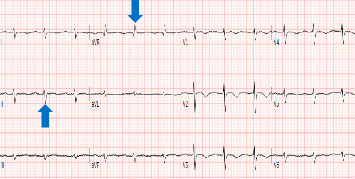
Twelve lead surface electrocardiogram with low voltage of QRS complexes (blue arrows) and prolonged corrected QT interval measuring 506 ms.

**Figure 3:**
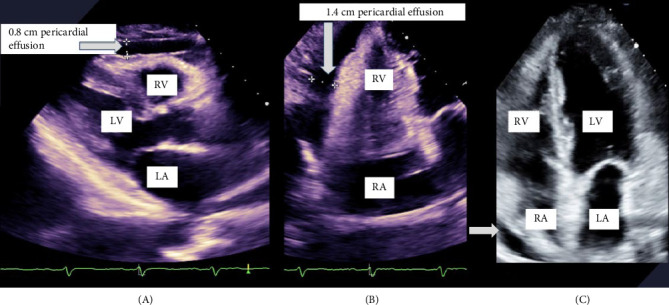
Electrocardiogram gated transthoracic echocardiogram showing a small localized pericardial effusion anterior and lateral to right side. (A) Left parasternal view: left atrium (LA), left ventricle (LV), right ventricle (RV), and 0.8 cm pericardial effusion anterior to RV (gray arrow). (B) Apical four chamber view focused on lateral side of RV with 1.4 cm pericardial effusion lateral to RV (gray arrow). (C) Apical four chamber view: right atrium (RA), RV, LA, and LV. Small pericardial effusion lateral to RA (gray arrow).

She reported chronic constipation and reduced functional capacity. She has four children, with last pregnancy 18 years ago, and complicated by postpartum hemorrhage that requires blood transfusion. On further questioning, she recalled being unable to lactate, with absence of menstrual cycles thereafter. She has not had medical evaluation before this presentation. Laboratory evaluation while on IV pressors and stress steroids revealed hypo-osmolar hyponatremia with low urine osmolality and sodium, as well as hyperlipidemia (Tables 1–3). Pituitary hormone testing confirmed central hypothyroidism with undetectable free thyroxine (FT4) and normal TSH, low follicle-stimulating hormone (FSH) and luteinizing hormone (LH), estradiol, insulin-like growth factor-1 (IGF-1), and prolactin (PRL) (Table 4). Brain magnetic resonance imaging (MRI) performed for altered mental status evaluation showed a partial empty sella (Figure 1). Further testing revealed low-voltage complexes and prolonged QTc on electrocardiogram (ECG) (Figure 2), a small pericardial effusion, and normal ventricular function on transthoracic echocardiogram (TTE) (Figure 3).

IV thyroxine dose of 200 microgram (mcg) was started, followed by 75 mcg/day Empirical IV antibiotics were initiated until sepsis was excluded. Workup for anemia showed a normal iron profile, vitamin B12, and folate levels.

**Figure 4:**
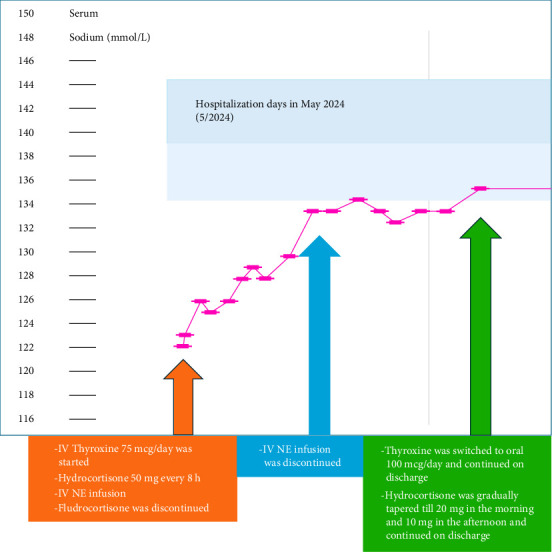
Serum sodium on the vertical axis in mmol/L and hospitalization days in May 2024 on the horizontal axis. Initial low serum sodium of 122 mmol/L on 5/3/2024 that improved after starting intravenous (IV) thyroxine (T4), then IV levothyroxine was switched to oral 100 mcg/day on 5/6/2024. IV norepinephrine (NE) infusion discontinued after 2 days on 5/5/2024. Stress hydrocortisone dose was tapered gradually. Orange arrow points serum sodium (122 mmol/L) on initial presentation and orange rectangle includes the medications that were given. Blue arrow points serum sodium 2 days after presentation and blue rectangle outlines medication change. Green arrow points serum sodium (136 mmol/L) after 3 days and green rectangle outlines the medications change.

The patient improved significantly with IV thyroxine. She resumed bowel movements, became more interactive, and exhibited improved speech. Serum sodium normalized from 122 to 136 millimole per liter (mmol/L), and IV NE was discontinued after 2 days. Stress-dose steroids were gradually tapered to oral HC 20 mg in the morning and 10 mg in the afternoon. After 3 days, she was transitioned to oral thyroxine at 100 mcg/day (Figure 4). She was discharged on oral thyroxine and HC with endocrinology follow-up for further hormonal evaluation and long-term management.

## 3. Discussion

MC is a rare and lethal complication of Sheehan's syndrome [1]. Central hypothyroidism is less common than primary hypothyroidism, with an estimated prevalence of 1 in 20,000 to 1 in 80,000 [4]. A normal TSH level alone is insufficient to exclude thyroid disorders, as failure to assess FT4 can delay or miss the diagnosis of central hypothyroidism. Our case has panhypopituitarism secondary to Sheehan's syndrome with an 18-year delay in diagnosis and management. Clinical features of myxedema were noted with normal TSH which raises the possibility of central hypothyroidism. Subsequent hormonal evaluation confirmed the diagnosis with an undetectable free T4 and inappropriately normal TSH. Further workup revealed panhypopituitarism including central hypogonadism (low FSH, LH, and undetectable estradiol associated with secondary amenorrhea), low IGF-1, and low PRL levels.

We concluded that MC was due to prolonged untreated central hypothyroidism after exclusion of septic and circulatory causes. Our case exhibited other features of hypothyroidism, including a small pericardial effusion, low QTc voltage, normocytic normochromic anemia, and hyperlipidemia, which may also be attributed to deficiencies in other pituitary hormones. Neutrophilia, lymphopenia, and eosinopenia could be due to steroid therapy. Although hypoglycemia and bradycardia may have been masked by IV pressors and stress steroid, the hypotension, hypothermia, and hyponatremia only improved following IV thyroxine administration. The duration of panhypopituitarism prior to presentation is unclear; however, the history of postpartum hemorrhage, failure of lactation, and secondary amenorrhea strongly suggests long-standing hypopituitarism due to pituitary apoplexy.

The causes of central hypothyroidism include structural lesions, trauma, surgery, hemorrhage, ischemia, irradiation, inflammation, infiltration, or genetic disorders of the pituitary gland and/or hypothalamus [2]. More recently, immune checkpoint inhibitors, particularly cytotoxic T-lymphocyte-associated antigen 4 (CTLA-4) inhibitors, have been recognized as an emerging cause of hypophysitis [5]. Sheehan's syndrome results from postpartum hemorrhage leading to pituitary infraction. While its incidence has declined due to advances in obstetric care, cases with a remote history of postpartum hemorrhage may still be diagnosed years later through a detailed history, often after presenting with central hormone deficiencies [1], as seen in our case. Hyponatremia is frequently associated with hypothyroidism, particularly MC and AI. It is often attributed to a syndrome of inappropriate antidiuretic hormone secretion (SIADH)-like state, thought to result from impaired renal perfusion and reduced free water excretion, which improves with hormonal replacement therapy [6].Our patient presented with hypo-osmolar hyponatremia, but low urine osmolality and specific gravity were inconsistent with SIADH. Additionally, low urine sodium could be due to dehydration. It is noteworthy that urinary evaluation was performed, while the patient was receiving IV pressors and stress steroid. Serum sodium improved to 136 mmol/L with IV isotonic saline and IV thyroxine.

Diagnosing MC is challenging and requires a high index of clinical suspicion. A scoring system has been proposed, where a score >60 strongly suggests MC [7]. Retrospectively, our patient had a score of 65. Early recognition and treatment of MC are critical to improve the survival rates. The American Thyroid Association (ATA) recommends administering stress-dose steroids before initiating IV thyroxine therapy, with a loading dose of 200–400 mcg IV thyroxine, followed by a weight-based maintenance dose of 1.6 mcg/kg/day orally or 75% of the calculated dose if given IV [8]. Routine triiodothyronine (T3) therapy is not recommended due to its higher potency and risk of cardiac ischemia. However, T3 can be added to levothyroxine in cases of persistent hypothyroid symptoms or in patients with malabsorption syndromes affecting levothyroxine uptake [9].

The goal of CH treatment is to maintain FT4 levels in the upper half of the normal reference range. A persistently unsuppressed TSH may indicate undertreatment [2]. When initiating hormone replacement therapy, interactions between thyroxine and other hormones should be considered. Estrogen therapy increases thyroid-binding globulin (TBG), requiring a higher thyroxine dose. Testosterone replacement reduces TBG, potentially lowering thyroxine requirements and higher doses of thyroxine are needed with growth hormone replacement [2].

## 4. Conclusion

Central hypothyroidism can be overlooked when relying solely on TSH screening without measuring FT4. Sheehan's syndrome is now rare; however, it should be considered during history taking for central endocrine disorders.

## Figures and Tables

**Table 1:** Initial laboratory workup.

Laboratory workup	Result	Normal range
Serum sodium	122 mmol/L	135–145 mmol/L
Serum potassium	4.7 mmol/L	3.5–5.2 mmol/L
Blood urea nitrogen	5 mg/dL	6–20 mg/dL
Creatinine	0.76 mg/dL	0.4–1.2 mg/dL
Glucose	210 mg/dL	—
Glomerular filtration rate	99 mL/min/1.73 m^2^	> 60 mL/min/1.73 m^2^
Hemoglobin	8.8 g/dL normocytic normochromic	11.7–16 g/dL
White blood cells	19.7 k/μL	3.9–12 k/μL
Absolute neutrophils	18.3 k/μL	1.3–7.5 k/μL
Absolute eosinophils	0.0 k/μL	0.0–0.5 k/μL
Absolute lymphocyte	1.2 k/μL	1.3–4.2 k/μL
Platelets	265 k/μL	150–450 k/μL
Aspartate aminotransferase	40 U/L	10–40 U/L
Alanine aminotransferase	16 U/L	7–50 U/L
Alkaline phosphatase	93 U/L	40–125 U/L
Hemoglobin A1C	5%	<5.7%
TSH (thyroid-stimulating hormone)	0.96 mIU/mL	0.3–4 mIU/mL

*Note:* Initial laboratory work up remarkable for hyponatremia, normocytic normochromic anemia, leukocytosis, neutrophilia, lymphopenia, and eosinopenia. Patient was receiving intravenous norepinephrine infusion and hydrocortisone 50 mg every 8 h and oral fludrocortisone 0.1 mg/day. mL/min/1.73 m^2^, milliliters of cleansed blood per minute per body surface.

Abbreviations: %, percentage; g/dL, gram per deciliter; k/μL, kilo per microliter; mg/dL, milligrams per deciliter; mIU/mL, milli-international units per liter; moml/L, millimoles per liter; U/L, unit/liter.

**Table 2:** Lipid profile.

Lipid profile	Result	Normal range
Low density lipoprotein (LDL)	211 mg/dL	<130 mg/dL
Triglycerides	89 mg/dL	<150 mg/dL
High density lipoprotein (HDL)	34 mg/dL	>34 mg/dL
Total cholesterol (TC)	263 mg/dL	<200 mg/dL

*Note:* Remarkable for high LDL and TC and low HDL.

Abbreviation: mg/dL, milligrams per deciliter.

**Table 3:** Initial laboratory workup.

Laboratory workup	Result	Normal range
Serum osmolality	270 mosm/kg	279–300 mosm/kg
Urine osmolality	121 mosm/kg	300–900 mosm/kg
Urine specific gravity	1005	1003–1035
Urine sodium	<10 mmol/L	—
Urine creatinine	26 mg/dL	—

*Note:* Remarkable for low serum and urine osmolality. Low urine specific gravity and urine sodium. Patient was receiving intravenous norepinephrine infusion and hydrocortisone 50 mg every 8 h and oral fludrocortisone 0.1 mg/day.

Abbreviations: mg/dL, miligram per deciliter; mmol/L, millimole per liter; mosm/kg, milliosmoles per kilogram.

**Table 4:** Hormonal workup.

Hormone workup	Result	Normal range
Free thyroxine (FT4)	<0.3 ng/dL	0.6–1.7 ng/dL
Total triiodothyronine (T3)	28 ng/dL	80–178 ng/dL
TSH (thyroid-stimulating hormone)	0.96 mIU/mL	0.3–4 mIU/mL
Follicle-stimulating hormone (FSH)	1.5 mIU/mL	Follicular phase: 3.4–10.0; luteal phase: 1.9–5.1; midcycle: 5.7–20.0; postmenopausal: 41.0–129.0
Luteinizing hormone (LH)	0.3 mIU/mL	Follicular phase: 2.1–10.9; luteal phase: 1.2–12.9; midcycle: 19.2–103.0; postmenopausal: 10.9–58.6
Estradiol	<15 pg/mL	Follicular phase: 25–115; luteal phase: 36–246; midcycle: 32–517; postmenopausal: 15–25 pg/mL
Prolactin	1.8 ng/mL	3.3–26.7 ng/mL
Insulin growth factor-1 (IGF-1)	<10 ng/mL	66–249 ng/mL

*Note:* Hormonal work up is remarkable for undetectable FT4, normal TSH, and low FT4, FSH, LH, estradiol, IGF-1, and prolactin. Consistent with central hypothyroidism, central hypogonadism, and adult growth hormone deficiency. We could not assess adrenal axis as patient was on steroids, but she has had low random cortisol in another facility before starting steroids. Patient was receiving on intravenous norepinephrine infusion and hydrocortisone 50 mg every 8 h and fludrocortisone 0.1 mg/day.

Abbreviations: mIU/mL, milli-international units per milliliter; ng/dL, nanogram per deciliter; pg/mL, picogram per mililiter.

## Data Availability

All data generated or analyzed during this study are included in this published article. Further inquiries can be directed to the corresponding author.
